# A Novel Multi-Sensor Fusion Algorithm Based on Uncertainty Analysis

**DOI:** 10.3390/s21082713

**Published:** 2021-04-12

**Authors:** Haobai Xue, Maomao Zhang, Peining Yu, Haifeng Zhang, Guozhu Wu, Yi Li, Xiangyuan Zheng

**Affiliations:** 1 Tsinghua Shenzhen International Graduate School, Tsinghua University, Shenzhen 518055, China; xue.haobai@sz.tsinghua.edu.cn (H.X.); liyi@sz.tsinghua.edu.cn (Y.L.); zheng.xiangyuan@sz.tsinghua.edu.cn (X.Z.); 2 Shenzhen Institute of Information Technology, Shenzhen 518172, China; peining.yu@sziit.edu.cn; 3 Research Institute of Tsinghua, Pearl River Delta, Guangzhou 510700, China; zhanghf@tsinghua-gd.org; 4 Shenzhen LeEngSTAR Technology Co. Ltd., Shenzhen 518055, China; wuguozhu@leengstar.com

**Keywords:** uncertainty analysis, Monte Carlo, two-phase flow, multi-sensor fusion, electrical capacitance tomography, differential pressure, Venturi, cross-correlation

## Abstract

During the research and development of multiphase flowmeters, errors are often used to evaluate the advantages and disadvantages of different devices and algorithms, whilst an in-depth uncertainty analysis is seldom carried out. However, limited information is sometimes revealed from the errors, especially when the test data are scant, and this makes an in-depth comparison of different algorithms impossible. In response to this problem, three combinations of sensing methods are implemented, which are the “capacitance and cross-correlation”, the “cross-correlation and differential pressure” and the “differential pressure and capacitance” respectively. The analytical expressions of the gas/liquid flowrate and the associated standard uncertainty have been derived, and Monte Carlo simulations are carried out to determine the desired probability density function. The results obtained through these two approaches are basically the same. Thereafter, the sources of uncertainty for each combination are traced and their respective variations with flowrates are analyzed. Further, the relationship between errors and uncertainty is studied, which demonstrates that the two uncertainty analysis approaches can be a powerful tool for error prediction. Finally, a novel multi-sensor fusion algorithm based on the uncertainty analysis is proposed. This algorithm can minimize the standard uncertainty over the whole flowrate range and thus reduces the measurement error.

## 1. Introduction

Oil and nature gas are critical strategic resources that support the national economy and people’s livelihood, and their exploration, extraction, transportation and processing all involve the measurement of multiphase flow [1]. Therefore, it is of great significance to accurately measure its flowrate [2]. Currently, the commonly used metering method is to separate the multiphase flow into oil, gas and water first and then measure their respective flowrates with single-phase flowmeters [3]. However, the separators are usually bulky and expensive, and the separation process is time-consuming [4]. Therefore, real-time online measurements for each individual oil well cannot be realized with this method. In addition, with the depletion of onshore oil fields, more attentions have been turned offshore, where the compact and expensive offshore platforms place more stringent requirements for the size of a multiphase metering system [3,4].

In order to solve the above-mentioned technical problems, multi-phase flowmeters (MPFMs) using the combination of a Venturi tube and a gamma-ray densitometer have been proposed [5,6]. The attenuation rate of gamma-ray varies with the media density, which is employed by the densitometer to estimate the mixture density [7,8]. However, the radioactive sources are harmful to both the environment and human bodies, and their production and application usually requires approval from local authorities, which leads to higher maintenance costs. Therefore, non-separative, non-radioactive and non-intrusive multiphase flowmeters are under development by different companies [9,10], and the most commonly deployed techniques include the cross-correlation [11,12,13,14,15], the differential pressure meters [16,17], and the Electrical Capacitance Tomography (ECT) [18,19,20,21,22]. As for the gas-oil two-phase flow, the combinations of any two of the above three sensing methods can be implemented to determine the gas and liquid flowrates, which are referred to as the “capacitance and cross-correlation” (“Cap + CC”), the “cross-correlation and differential pressure” (“CC + DP”), and the “differential pressure and capacitance” (“DP + Cap”) respectively. All of these three combinations have been extensively studied in the literature [23,24,25,26,27,28,29]. For example, a dual-plane electrical resistance tomography (ERT) system for gas-liquid flow measurement has been studied by Dong [23,24]. The combination of cross-correlation and Venturi meter has been proposed by Harstad [28] and Fueki [25]. A Venturi meter and electrical capacitance tomography (ECT)/ERT system for gas-liquid flow measurement has been analyzed by Huang [26,27] and Meng [29]. However, a fair comparison of the above-mentioned combinations remains largely absent because the input data, benchmarking data and model predictions are generally different and the relevant uncertainty information is usually lacking. Besides, errors rather than uncertainties are often used to evaluate the performance of different algorithms, but the errors can only be determined when the true values (or reference values) are known and their values often possess a certain degree of randomness, which makes it difficult to trace their sources. All these characteristics make the research centered on errors somewhat limited, and an in-depth comparison of different algorithms impossible.

The studies on the uncertainty analysis of multiphase flow meters are relatively rare but still exist. For example, the limitations imposed by the theoretical models for multiphase flow metering were first discovered by Millington [30], and he concluded that uncertainty data must be qualified with a statement of the flow composition to which it applies. Later, a simple uncertainty analysis (UA) of the gas-oil two-phase flow was presented by Kouba [31] and the contour maps of oil flowrate uncertainty with respects to the gas fraction and water cut were provided. It was concluded that UA is a valuable, but often overlooked, tool for multiphase metering systems. The measurement uncertainty of vortex flowmeter was examined by Jia et al. [32] when a small amount of liquid is injected into the gas flow. The different components of the total uncertainty are analyzed, and the dominant one is specially treated and descended by increasing the sampling number. The characterization of confidence in multiphase flow predictions was reviewed by Cremaschi et al. [33], with the aim to raise the awareness of the importance of UA and to identify some of the key gaps in the UA. The field experiences and challenges with regard to quantifying measurement uncertainties were summarized and presented by Folgerø et al. [34], and it was found that the representativeness of reference measurement, fluid densities and production profiles are all factors that affected the uncertainty strongly. Recently, uncertainty analysis for horizontal air-water flow experiment was conducted by Jaloretto [35], and the flow pattern maps and two-phase pressure drops with their uncertainties were provided to better understand the flow pattern transition regions and the pipe diameter influence.

From the above literature review, it can be concluded that the importance of uncertainty analysis is generally acknowledged, and the facts that uncertainties vary with different flow conditions and total uncertainties can be divided into different components are well-known. However, from the author’s perspective, the researches on the following topics are still relatively insufficient.

1. The compositions of uncertainties

Multiphase flowmeters do not measure the individual flowrates directly, but infer them from a collection of indirect measurements [30], such as differential pressure, time delay of cross-correlation and average capacitance, etc. Therefore, uncertainty analysis can be a useful tool for tracing the sources of uncertainties, and thus provide guidance for more targeted system improvement. For example, if it is already known that the uncertainties of flowrates are dominated by the densitometer rather than the Venturi tube, then replacing the differential pressure sensor with a more accurate but more expensive one will not help improve the system performance significantly.

2. The distributions of uncertainties

Uncertainty analysis can help MPFM designer know in which flow conditions a certain flowrate algorithm underperforms and take actions to prevent it. For example, it is well known that an additional sensor will incur higher costs but not necessarily higher accuracy, especially if this additional sensor is not as accurate as others. Uncertainty analysis allows the system to compare the uncertainties of all possible algorithms and make sure the flow conditions one algorithm underperforms in are not used as the final outputs.

3. The connections between errors and uncertainties

It is well known that errors and uncertainties are related, and the ultimate goal of MPFM development is to reduce the measurement errors. However, an algorithm to reduce measurement errors through uncertainty minimization is still absent in the literature.

In this paper, two approaches of uncertainty analysis are carried out: the first one is to derive the associated uncertainty through the analytical expressions of the gas/liquid flowrate; and the second one is to determine the probability density function (PDF) of the gas/liquid flowrate output by the Monte Carlo simulations. The results obtained through these two approaches are basically the same, such as the flowrate estimates and standard uncertainty. Thereafter, the sources of uncertainty for each combination are traced, and their respective variations with flowrates are analyzed. Further, the relationship between error and uncertainty is studied, which demonstrates that uncertainty analysis can be a powerful tool for error prediction. Finally, a novel multi-sensor fusion algorithm based on uncertainty analysis is proposed. This algorithm can minimize the standard uncertainty over the whole range and thus reduces measurement errors, as well as making their distribution more even.

## 2. Metering System and Method

### 2.1. Multiphase Flow Test Facility

**Figure 1:**
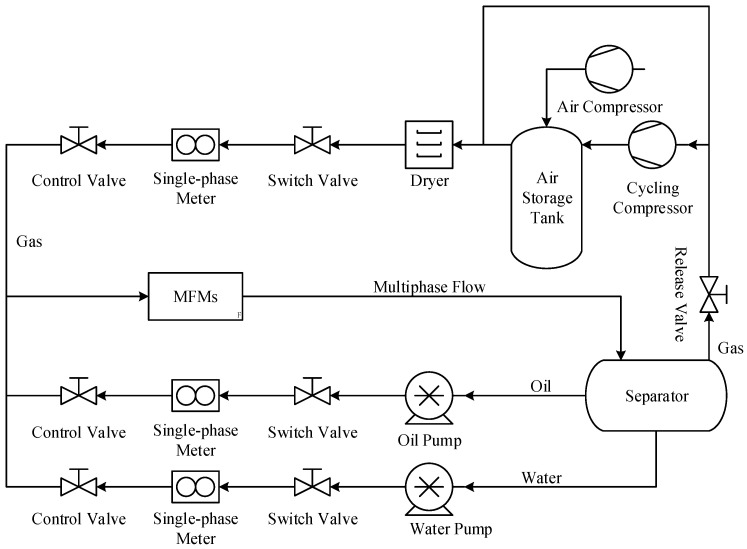
Schematic diagram of the multiphase flow experimental facility.

The schematic diagram of the multiphase flow test facility is shown in Figure 1. In order to simulate the working conditions of the MPFMs, the whole system is pressurized to 0.3 MPa by the air compressor first. Then the gas flow is supplied by the cycling compressor, whilst the oil and water flows are supplied by the oil pump and water pump respectively. The flowrate of the gas, oil and water are metered by a turbine flowmeter, a volumetric flowmeter and an electromagnetic flowmeter respectively. The control valves are used to adjust the flowrate of each single-phase flow and these single-phase flows are mixed before passing through the MPFM. Finally, the multiphase flow will be separated and reused in the separator.

### 2.2. MPFM

**Figure 2:**
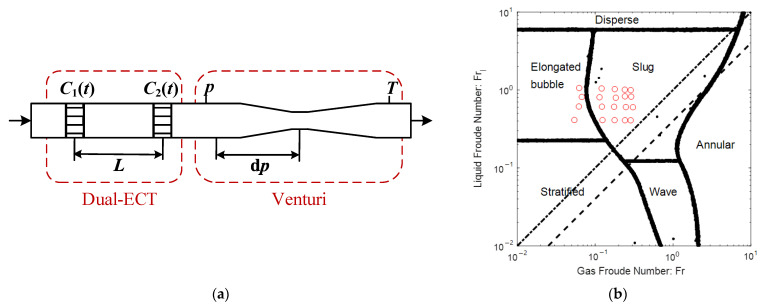
Schematic diagram of the multi-phase flowmeter (MPFM) and the collected test data: (**a**) MPFM; (**b**) test data.

The schematic diagram of the MPFM is shown in Figure 2a. The mixture of air and oil first flows through a high-frequency capacitance detection module (or dual-ECT module, for short), and then flows through a differential pressure flowmeter module (or Venturi module, for short).

The dual-ECT module includes two planes of ECT sensors separated at a distance of L=110 mm, and the detection frequency is f=714 Hz. Each plane of ECT sensor includes 8 electrodes, so that 28 independent capacitance measurements can be obtained for each detection. The Venturi module includes a Venturi tube to measure the differential pressure dp, and a pressure and temperature sensor to measure p and T respectively. The diameters of the Venturi inlet and throat are D=50 mm and d=25 mm respectively, so that the diameter ratio is β=d/D=0.5. The detection frequency of the Venturi module is f=10 Hz.

The 20 test points are shown as red dots in Figure 2b, from which it is notable that the flow patterns are mainly slug flow and elongated bubble flow. The test pressure is 0.3 MPa, and the liquid and gas flowrate ranges are $2\sim 5{\;m}^{3}/h$ and $15\sim 75{\;\mathrm{Nm}}^{3}/h$ respectively.

#### 2.2.1. Dual-ECT

The measured capacitance $C_{i}$ can be used to determine the Liquid Volume Fraction LVF through linear data fitting. The two time-series signals of capacitance $C_{1}\left(t\right)$ and $C_{2}\left(t\right)$ can be used to estimate the total volume flowrate through cross-correlation as follows [2,3,4]:

(1) $$R_{C_{1},C_{2}}\left(\tau \right)=\;\underset{T\to \infty }{\lim}\frac{1}{T}\overset{T}{\underset{0}{\int }}C_{1}\left(t\right)C_{2}\left(t+\tau \right)dt$$

The discrete form of the above equation can be written as:

(2) $$R_{C_{1},C_{2}}\left(jdt\right)=\;\frac{1}{N}\overset{N}{\underset{n=1}{\sum }}C_{1}\left(n\right)C_{2}\left(n+j\right)\;j=0,1,2,\cdots$$

When $j=j_{\max}$, the cross-correlation function $R_{C_{1},C_{2}}\left(jdt\right)$ reaches its maximum, and thus the time delay $\tau_{\max}$ can be calculated from $\tau_{\max}=j_{\max}dt$. $\tau_{\max}$ is the estimate of the time when the fluid passes from the upstream to the downstream sensor, so the fluid velocity $u_{th}$ can be estimated by $u_{th}=L/\tau_{\max}$ and the estimated total flowrate $Q_{th}$ can be estimated by:

(3) $$Q_{th}=A_{d}\frac{L}{\tau_{\max}}$$

where $A_{d}$ is the cross-sectional area of the pipe.

#### 2.2.2. Venturi

It should be noted that the flowrate equation provided by ISO 5167-4 [36] is not suitable for multiphase flow working conditions, so that the obtained flowrate is referred to as the indicated gas flowrate $Q_{tp}$ which is not equal to the actual gas flowrate $Q_{g}$.

(4) $$Q_{tp}=\frac{C_{d}\varepsilon A}{\sqrt{1-\beta^{4}}}\sqrt{\frac{2dp}{\rho_{g}}}$$

where A denotes the cross-sectional area of the Venturi throat, dp is the measured pressure difference, β is the diameter ratio, β=d/D, $C_{d}$ and ε are the discharge coefficient and the expansion factor respectively, and both of them can be determined by ISO 5167-4 [36]. $\rho_{g}$ denotes the gas density, which can be calculated from the measured pressure p and temperature T according to ISO 12213-2 [37].

### 2.3. Uncertainty Analysis Methods

One of the most fundamental and comprehensive documents for uncertainty analysis is the “guide to the expression of uncertainty in measurement” (hereinafter referred to as the GUM method) [38] and its supplement 1 “Propagation of distributions using a Monte Carlo method” (hereinafter referred to as the MCM method) [39].

#### 2.3.1. GUM

The basic idea of the GUM method is that the model input information can be expressed in the forms of its estimate and standard uncertainty, and these estimates and standard uncertainties are propagated through a (linearized) model to provide the estimate and standard uncertainty of the model output. Normal distributions are assumed for the model output according to the Central Limit Theorem; therefore, the coverage factor and expanded uncertainty can be determined accordingly [40].

The general method to evaluate the input uncertainty through linear regression and the method to determine the output uncertainty through the law of propagation are summarized in Appendix A. More information about the GUM method can be found in Refs. [38,40].

#### 2.3.2. MCM

The MCM method (Monte Carlo method), also known as the statistical simulation method or random sampling method, is a method of using random numbers (or pseudo-random numbers) to solve problems. It randomly generates a value from the probability density function (PDF) of each input quantity and calculates its corresponding output value. This process is repeated many times, so that a total of M outputs can be obtained. The PDF and associated statistics can be determined from these M outputs.

In this paper, the level of confidence is set as p=0.95 and the number of simulations is set as $M=10^{6}$. The pseudo-random number generator is provided by Matlab (e.g., “random” and “mvnrnd” commands) and relevant codes can be directly obtained from Ref. [41]. It is worth mentioning that multi-variable normal distribution generators should be applied for the coefficients of the linear fitting ($\beta_{0}$ and $\beta_{1}$ in Equation (9)) because these coefficients are correlated so that their covariance must be taken into consideration. More information about the MCM method can be found in Refs. [39,41].

### 2.4. The Relationship between Certain Concepts

#### 2.4.1. Composition and Distribution Diagrams

**Figure 3:**
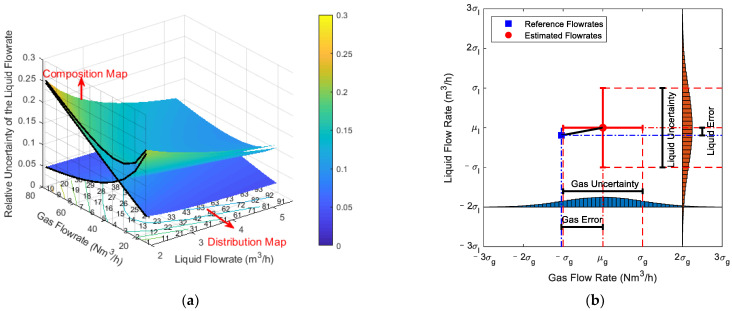
Schematic diagrams of the relationships between certain concepts: (**a**) the relationship between the composition and distribution diagrams; (**b**) the relationship between the error and uncertainty.

Figure 3a simultaneously shows the composition and distribution diagrams of the liquid flowrate uncertainty. It is notable from Figure 3a that the combined uncertainty varies with the gas and liquid flowrates, and the combined uncertainty is mainly made up of two components, which corresponds to the contributions from two different sensors.

(5) $$u_{c}\left(y\right)=\sqrt{u_{1}^{2}\left(y\right)+u_{2}^{2}\left(y\right)}$$

By cross-referring the composition and distribution diagrams in Section 3, it is possible to mentally construct similar 3-D diagrams as Figure 3a. The points numbered from 1 to 100 refers to 100 interpolated points that can help the readers accurately figure out the composition and distribution of certain typical working conditions.

With the distribution maps, the MPFM developers can develop novel algorithms that can minimize the total uncertainties in any gas and liquid flowrate conditions. With the composition maps, the MPFM developers can figure out the major sources of the uncertainties and carry out more targeted improvements. Relevant information can be found in the prediction result figures in Section 3.

#### 2.4.2. Error and Uncertainty

According to the GUM, the definitions of error and uncertainty are as follows:

- Error (of measurement). Result of a measurement minus a true value of the measurand.
- Uncertainty (of measurement). Parameter associated with the result of a measurement that characterizes the dispersion of the values that could reasonably be attributed to the measurand.

The difference between error and uncertainty is shown in Figure 3b. It is notable from the definitions of error and uncertainty that as long as the measured value and the true value (or reference value) of a measurand are known, its error is a definite number with a plus or minus sign; whereas its uncertainty is always a positive number. More detailed descriptions of the difference between error and uncertainty can be found in Refs. [38,40].

For each measurement, there is an associated error and uncertainty. Therefore, it is sometimes more convenient to use Mean Absolute Error (MAE) or Mean Absolute Percentage Error (MAPE) to compare the average values of errors of different data sets. The definitions of MAE and MAPE are as follows:

(6) $$\mathrm{MAE}=\frac{1}{n}\overset{n}{\underset{i=1}{\sum }}\left|{\hat{y}}_{i}-y_{i}\right|$$

(7) $$\mathrm{MAPE}=\frac{100\%}{n}\overset{n}{\underset{i=1}{\sum }}\left|\frac{{\hat{y}}_{i}-y_{i}}{y_{i}}\right|$$

where ${\hat{y}}_{i}$ is the measured value, $y_{i}$ is the reference value and n is the number of measurements of a dataset. The ranges of MAE of MAPE are both from 0 to infinity and larger MAE and MAPE mean lower accuracy. But MAE is usually used for the errors of ratios (e.g., LVF) whereas MAPE is mainly used for the errors of flowrates (e.g., $Q_{th}$ and $Q_{tp}$). In the following part of this paper, the relative error in the figure titles refers to the MAPE.

Similar terms such as Mean Absolute Uncertainty (MAU) or Mean Absolute Percentage Uncertainty (MAPU) can be defined for the standard uncertainties of a dataset. In the following part of this paper, the relative uncertainty in the figure titles refers to the MAPU.

The ultimate goal of MPFM development is to reduce its measurement error and it is well known that the error and uncertainty are related. Therefore, it is worth developing new algorithm that can reduce the measurement errors through careful uncertainty minimizations, and relevant information can be found in Section 3.

### 2.5. Expanded Uncertainty

Expanded uncertainty defines an interval of the measurement result to which a certain level of confidence can be attributed. It is obtained by multiplying the combined standard uncertainty $u_{c}\left(y\right)$ by the coverage factor k:

(8) $$U\left(y\right)=ku_{c}\left(y\right)$$

According to the Central Limit Theorem, normal distributions are assumed for both inputs and outputs, and the degree of freedom will be sufficiently high because the direct measurements such as differential pressure and capacitance are usually averaged over an extended period of time and the sampling frequency is relatively high. Therefore, $k_{95}=1.96$ can be used as the coverage factor for $U_{95}\left(y\right)$ with a level of confidence of 95%.

For the MCM method, if the simulation results show that the distributions of outputs deviate from the normal distribution, then a more accurate numerical method for estimating the interval $\left[y_{min},\;y_{max}\right]$ can be used. For example, if the test number is $M=10^{6}$ and the level of confidence is p=0.95, then the interval $\left[y_{min},\;y_{max}\right]$ can be determined by seeking the shortest interval that covers $pM=0.95\times 10^{6}$ test results. Details of this method can be found in Ref. [41] and discussions of this case can be found in Section 3.2.

It is worth mentioning that the intention of this paper is not to provide a comprehensive and detailed uncertainty analysis for all algorithms, but to use uncertainty analysis as a tool for composition and distribution analysis, and measurement error reduction, as emphasized in the introduction. In this sense, the standard uncertainty fulfills this function well and the extended uncertainty can simply be estimated by multiplying it with a constant (e.g., 2 for a rough estimate). Therefore, only the standard uncertainty results will be presented in the following part of this paper to avoid redundancy.

## 3. Three Sensing Combinations

### 3.1. “Cap + CC” Method

#### 3.1.1. The Calculation Procedures

**Figure 4:**
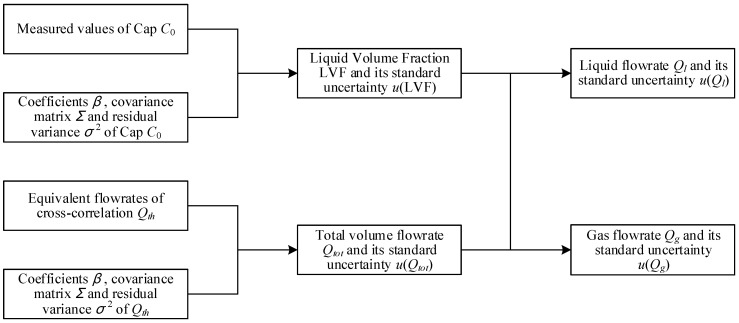
The calculation procedures of the capacitance and cross-correlation (Cap + CC) method.

The calculation process of the “Cap + CC” method is shown in Figure 4. From Figure 4, it can be noted that the total volume flowrate $Q_{\mathrm{tot}}$ and the liquid volume fraction LVF are both obtained through linear regression fitting. The average capacitance $C_{0}$ used for determining the LVF is obtained by averaging the capacitance of four opposite electrodes $C_{0}=\sum_{m=1}^{4}C_{m}/4$, whilst the equivalent flowrate of cross-correlation $Q_{th}$ used for determining the $Q_{\mathrm{tot}}$ is obtained by first averaging the time delays of eight adjacent electrodes $\tau_{th}=\sum_{n=1}^{8}\tau_{Cn}/8$, and then calculating the equivalent flowrate $Q_{th}$ through the following equation: $Q_{th}=AL/\tau_{th}$.

Thereafter, the average capacitance $C_{0}$ and the equivalent flowrate of cross-correlation $Q_{th}$ are used as the dependent variable y, whilst the reference values of LVF and $Q_{tot}$ are used as the independent variable x, and linear regression fittings are conducted in the following form:

(9) $$y=\beta_{0}+\beta_{1}x$$

**Figure 5:**
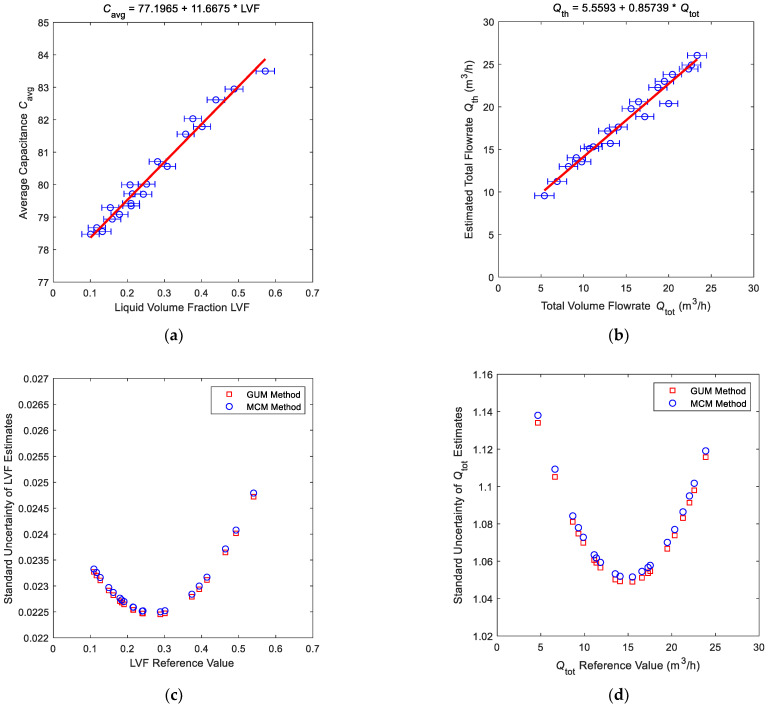
The fitting results and standard uncertainty distributions of important intermediate variables of the “Cap + CC” method: (**a**) the fitting results of the liquid volume fraction (LVF); (**b**) the fitting results of the total volume flowrate $Q_{tot}$; (**c**) the standard uncertainty distributions of the liquid volume fraction LVF; (**d**) the standard uncertainty distributions of the total volume flowrate $Q_{tot}$.

The linear fitting result of the $C_{0}$ and LVF is shown in Figure 5a, and the linear fitting result of the $Q_{th}$ and $Q_{tot}$ is shown in Figure 5b. The coefficient $\hat{\beta }$, the covariance matrix $\Sigma_{\hat{\beta }\hat{\beta }}$ and the standard uncertainty of dependent variable u(y) can be calculated by Equations (A3)–(A5) respectively.

According to the calculation process in Figure 4, the estimates of ${\mathrm{LVF}}_{0}$ and $Q_{tot0}$ should be calculated from the $C_{0}$ and $Q_{th}$ respectively. The used equation is simply $x_{0}=\left(y_{0}-\beta_{0}\right)/\beta_{1}$, and the associated standard uncertainty $u\left(x_{0}\right)$ is:

(10) $$u\left(x_{0}\right)=\frac{s}{\beta_{1}}\sqrt{\frac{1}{p}+\frac{1}{n}+\frac{{\left(x_{0}-\bar{x}\right)}^{2}}{S_{xx}}}$$

where $s=\sqrt{\frac{\sum_{i=1}^{n}v_{i}^{2}}{n-2}}$, $S_{xx}=\sum_{i=1}^{n}{\left(x_{i}-\bar{x}\right)}^{2}$, n is the data number of the training sets, p is the number of the repeated measurements of y.

The standard uncertainty distribution of the liquid volume fraction ${\mathrm{LVF}}_{0}$ is shown in Figure 5c, whilst the standard uncertainty distribution of the total volume flow $Q_{tot0}$ is shown in Figure 5d. It can be noted from Figure 5c,d and Equation (10) that the standard uncertainty of estimate $u\left(x_{0}\right)$ obtained from the calibration curve is related to the difference $\left(x_{0}-\bar{x}\right)$. If the estimate $x_{0}$ is close to the arithmetic mean $\bar{x}$, then the standard uncertainty of estimate $u\left(x_{0}\right)$ will be small, otherwise it will be large. On the other hand, this rule can also be used as the basis for reference point selection: the arithmetic mean $\bar{x}$ of the reference data set should be as close as possible to the point $x_{0}$ to be measured in the future.

After obtaining the estimates and standard uncertainty of LVF and $Q_{tot}$, the estimate and standard uncertainty of the liquid flowrate $Q_{l}$ can be calculated by the following equations:

(11) $$Q_{l0}=Q_{tot0}{\mathrm{LVF}}_{0}$$

(12) $$u_{rel}\left(Q_{l0}\right)=\sqrt{u_{rel}^{2}\left(Q_{tot0}\right)+u_{rel}^{2}\left({\mathrm{LVF}}_{0}\right)}$$

The estimate and standard uncertainty of the gas flowrate $Q_{g}$ can be calculated by similar equations with Equations (11) and (12).

#### 3.1.2. The Composition and Distribution Diagrams

**Figure 6:**
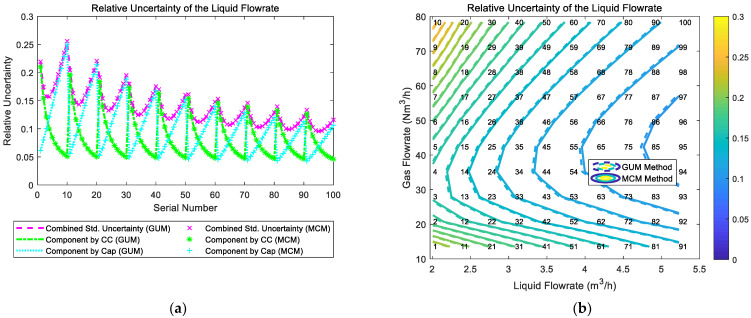
The composition and distribution of the relative uncertainty of the liquid flowrate of the “Cap + CC” method: (**a**) composition diagram; (**b**) distribution diagram. (Each contour line denotes an increment of uncertainty of 0.01. More information about the composition and distribution can be found in Section 2.4.1).

The composition and distribution of the relative uncertainty of the liquid flowrate of the “Cap + CC” method are shown in Figure 6. It can be noted from Figure 6a that the component of uncertainty introduced by the “CC” and the component of uncertainty introduced by the “Cap” are relatively similar. In addition, under certain liquid flowrate $Q_{l}$, with the gas flowrate $Q_{g}$ increasing, the component by “CC” gradually decreases whilst the component by “Cap” gradually increases, which causes the combined uncertainty of liquid flowrate $u_{rel}\left(Q_{l}\right)$ to decrease at first but increase later. With the liquid flowrate $Q_{l}$ increasing, the combined uncertainty of liquid flowrate $u_{rel}\left(Q_{l}\right)$ decreases monotonically. Therefore, the contour of the relative uncertainty $u_{rel}\left(Q_{l}\right)$ of the liquid flowrate of the “Cap + CC” method is shown in Figure 6b.

**Figure 7:**
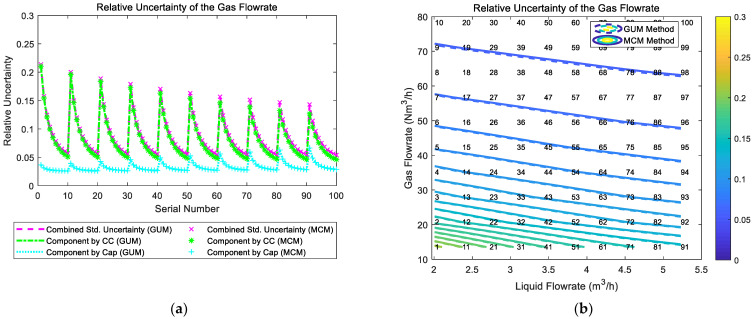
The composition and distribution of the relative uncertainty of the gas flowrate of the “Cap + CC” method: (**a**) composition diagram; (**b**) distribution diagram. (Each contour line denotes an increment of uncertainty of 0.01. More information about the composition and distribution can be found in Section 2.4.1).

The composition and distribution diagrams of the relative uncertainty of the gas flowrate of the “Cap + CC” method are shown in Figure 7. It can be noted from Figure 7a that the component of uncertainty introduced by “CC” is much larger than the component of uncertainty introduced by “Cap”, so the trend of the combined uncertainty of the gas flowrate $u_{rel}\left(Q_{g}\right)$ is dominated by its “CC” component. In addition, at certain liquid flowrate $Q_{l}$, with the gas flowrate $Q_{g}$ increasing, the components introduced by “CC” and “Cap” both decrease, which causes the combined uncertainty of the gas flowrate $u_{rel}\left(Q_{g}\right)$ to decrease monotonically. With the liquid flowrate $Q_{l}$ increasing, the combined uncertainty of the gas flowrate $u_{rel}\left(Q_{g}\right)$ also decreases monotonically. Therefore, the relative uncertainty of gas flowrate $u_{rel}\left(Q_{g}\right)$ of the “Cap + CC” method reaches its maximum at conditions with low liquid flowrate $Q_{l}$ and low gas flowrate $Q_{g}$, and its contour is shown in Figure 7b.

#### 3.1.3. The Connection between Error and Uncertainty

**Figure 8:**
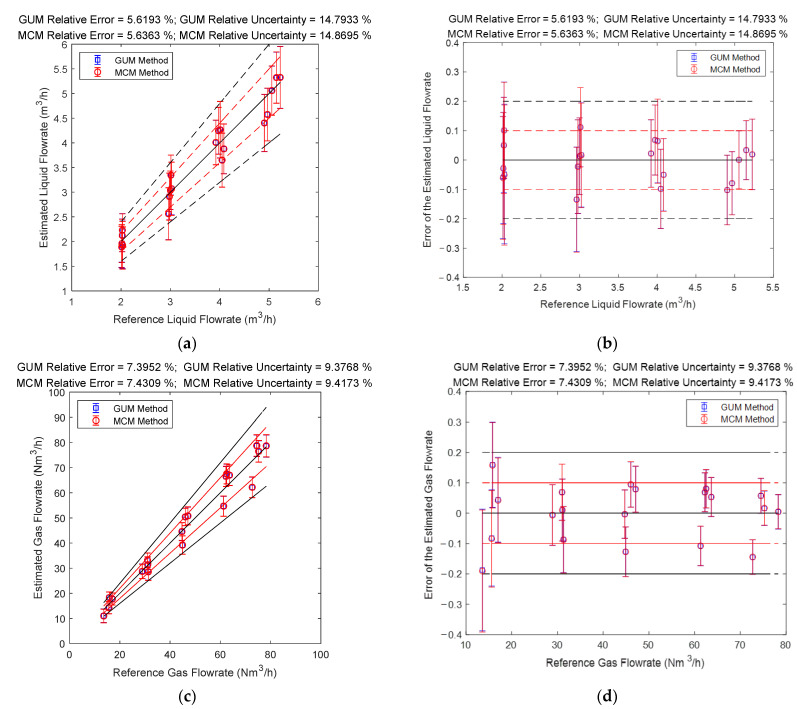
The prediction results of the liquid and gas flowrates of the “Cap + CC” method: (**a**) absolute error of the liquid flowrate; (**b**) relative error of the liquid flowrate; (**c**) absolute error of the gas flowrate; (**d**) relative error of the gas flowrate. (The central black line denotes the ideal case with zero error, the upper and lower red lines denote the 10% relative error range, and the upper and lower black lines denote the 20 % relative error range. The line segments denote the standard uncertainty whereas the distance between the point and the central black line denote the error. More information can be found in Section 2.4.2).

The gas and liquid flowrate prediction results of the “Cap + CC” method are shown in Figure 8. The blue lines in the Figure 8 denote the GUM results, whilst the red lines denote the MCM results. The central black line denotes the ideal case when the estimated value is equal to the reference value so that the error is always zero. The upper and lower red lines specify a 10% relative error range so that points within this range have relative errors less than 10%. Similarly, the upper and lower black lines specify a 20% relative error range. The line segments denote the standard uncertainty associated with a measurement and its value is represented by its length. The error is represented by the vertical distance between the test point and the central black line. More information about the distance between the error and uncertainty can be found in Section 2.4.2.

It can be noted from Figure 8 that the prediction results of the “Cap + CC” method for the liquid flowrate are relatively poor, while the prediction results for the gas flowrate are good, especially for working conditions with large gas flowrate. From Figure 8, it can be noted that the measurement error and standard uncertainty are related to each other: the longer the line segment is, the more likely the point will deviate from the central line. As shown in the titles of each figure, the larger the average standard uncertainty is, the larger the average relative error will likely become. Therefore, the standard uncertainty can be used for error prediction, thereby helping the operators know the accuracy of the results and providing assistance for better decision making.

### 3.2. “DP + Cap” Method

#### 3.2.1. The Calculation Procedures

**Figure 9:**
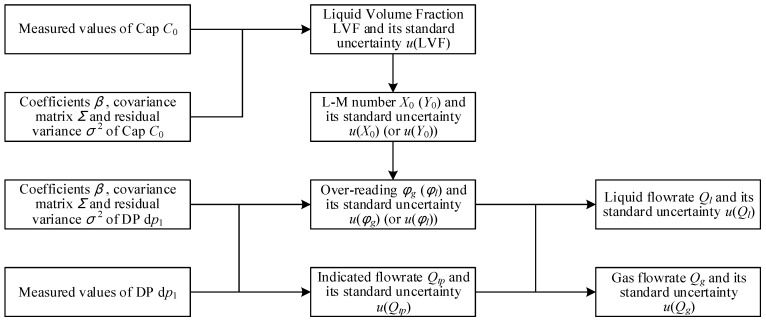
The calculation procedures of the differential pressure and capacitance (DP + Cap) method.

The calculation process of the “DP + Cap” method is shown in Figure 9. The method to calculate the estimate and standard uncertainty of the liquid volume fraction LVF is exactly the same as the “Cap + CC” method.

After ${\mathrm{LVF}}_{0}$ is obtained, it is then transformed into the L-M parameter $X_{0}$ by the following equation:

(13) $$X_{0}=\frac{{\mathrm{LVF}}_{0}}{1-{\mathrm{LVF}}_{0}}\sqrt{\frac{\rho_{l}}{\rho_{g}}}$$

According to the law of propagation of uncertainty, the standard uncertainty of the L-M parameter is:

(14) $$u\left(X_{0}\right)=\frac{u\left({\mathrm{LVF}}_{0}\right)}{{\left(1-{\mathrm{LVF}}_{0}\right)}^{2}}\sqrt{\frac{\rho_{l}}{\rho_{g}}}$$

Murdock [42], Bizon [43] and Lin [44] proposed a linear function between the gas over-reading $\phi_{g}=Q_{tp}/Q_{g}$ and the L-M parameter $X_{0}=\frac{Q_{l}}{Q_{g}}\sqrt{\frac{\rho_{l}}{\rho_{g}}}$ as follows:

(15) $$\phi_{g}=\beta_{0}+\beta_{1}X$$

If the above equation is multiplied by $Q_{g}$, then we have:

(16) $$Q_{tp}=\beta_{0}Q_{g}+\beta_{1}Q_{l}\sqrt{\frac{\rho_{l}}{\rho_{g}}}$$

where $Q_{tp}$ is the indicated gas flowrate, which is calculated by Equation (4).

**Figure 10:**
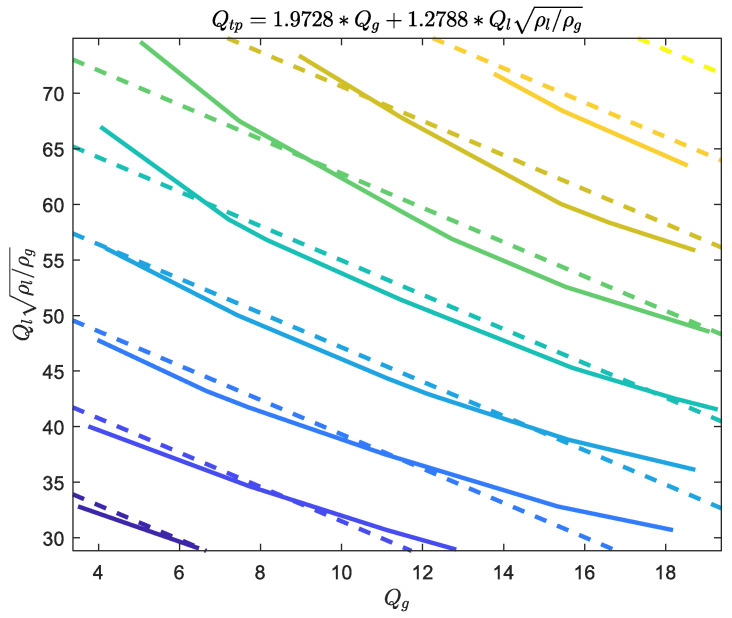
The fitting results of the indicated gas flowrate $Q_{tp}$ of the “DP + Cap” method.

In this paper, Equation (16) is used for the fitting of $Q_{tp}$ and the results are shown in Figure 10. The standard uncertainty $u\left(Q_{tp}\right)$ is thus considered as constant and its value can be calculated by Equation (A5). The coefficient $\hat{\beta }$ and covariance matrix $\Sigma_{\hat{\beta }\hat{\beta }}$ can be calculated by Equations (A3) and (A4) respectively. These parameters will be used later for determining the standard uncertainty of the gas/liquid flowrate.

It can be noted from Equation (16) that it can either be written as $Q_{tp}=Q_{g}\left(\beta_{0}+\beta_{1}X\right)=Q_{g}\phi_{g}$ or written as $Q_{tp}=Q_{l}\sqrt{\frac{\rho_{l}}{\rho_{g}}}\left(\beta_{0}Y+\beta_{1}\right)=Q_{l}\sqrt{\frac{\rho_{l}}{\rho_{g}}}\phi_{l}$. Therefore, as long as the estimates and standard uncertainties of the gas over-reading $\phi_{g}$ and liquid over-reading $\phi_{l}$ are known, then the estimates and standard uncertainties of the gas and liquid flowrate can be calculated from $Q_{g}=Q_{tp}/\phi_{g}$ and $Q_{l}=Q_{tp}\sqrt{\frac{\rho_{g}}{\rho_{l}}}/\phi_{l}$.

For example, the standard uncertainty of the gas over-reading $\phi_{g}$ can be calculated from:

(17) $$u\left(\phi_{g}\right)=\sqrt{s^{2}\left(\frac{1}{n}+\frac{{\left(x_{0}-\bar{x}\right)}^{2}}{S_{xx}}\right)+\beta_{1}^{2}u^{2}\left(X_{0}\right)}$$

If the correlation between $Q_{tp}$ and $\phi_{g}$ is negligible, then the uncertainty of gas flowrate $u_{rel}\left(Q_{g}\right)$ can be calculated by:

(18) $$u_{rel}\left(Q_{g}\right)=\sqrt{u_{rel}^{2}\left(Q_{tp}\right)+u_{rel}^{2}\left(\phi_{g}\right)}$$

The uncertainty of the liquid flowrate $u_{rel}\left(Q_{l}\right)$ can be calculated through similar equations as Equations (17) and (18)

#### 3.2.2. The Composition and Distribution Diagrams

**Figure 11:**
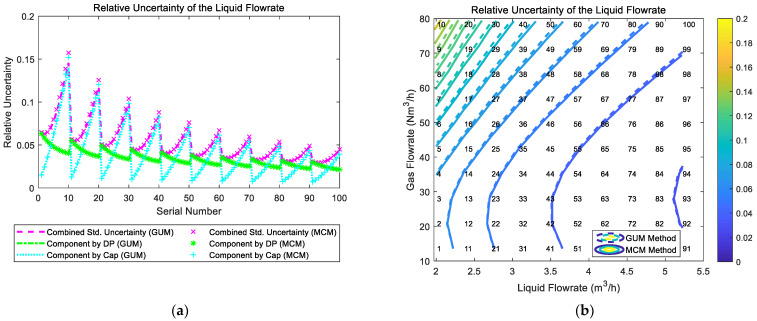
The composition and distribution of the relative uncertainty of the liquid flowrate of the “DP + Cap” method: (**a**) composition diagram; (**b**) distribution diagram. (Each contour line denotes an increment of uncertainty of 0.01. More information about the composition and distribution can be found in Section 2.4.1).

The composition and distribution of the relative uncertainty of the liquid flowrate of the “DP + Cap” method are shown in Figure 11. It can be noted from Figure 11a that the component of uncertainty introduced by “DP” and the component of uncertainty introduced by “Cap” are relatively similar. In addition, at certain liquid flowrate $Q_{l}$, with the gas flowrate $Q_{g}$ increasing, the component by “DP” gradually decreases, whilst the component by “Cap” gradually increases, which causes the combined uncertainty of the liquid flowrate $u_{rel}\left(Q_{l}\right)$ to slightly decrease at first and gradually increase then. With the liquid flowrate $Q_{l}$ increasing, the combined uncertainty of liquid flowrate $u_{rel}\left(Q_{l}\right)$ decreases monotonically. Therefore, the relative uncertainty of liquid flowrate $u_{rel}\left(Q_{l}\right)$ of the “DP + Cap” method reaches its maximum at working conditions with low liquid flowrate $Q_{l}$ and high gas flowrate $Q_{g}$, and its contour is shown in Figure 11b.

**Figure 12:**
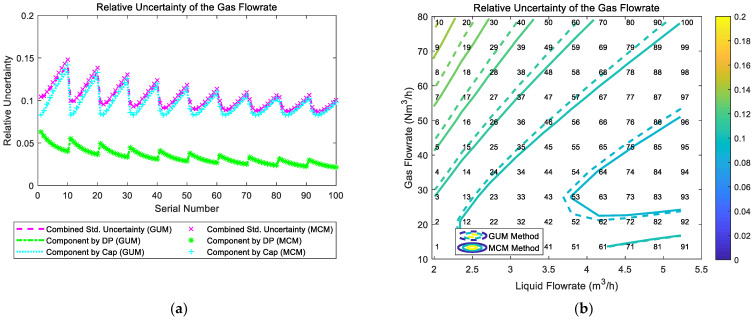
The composition and distribution of the relative uncertainty of the gas flowrate of the “DP + Cap” method: (**a**) composition diagram; (**b**) distribution diagram. (Each contour line denotes an increment of uncertainty of 0.01. More information about the composition and distribution can be found in Section 2.4.1).

The composition and distribution of the relative uncertainty of the gas flowrate of the “DP + Cap” method are shown in Figure 12. It can be noted from Figure 12a that the component of uncertainty introduced by “Cap” is much larger than the component of uncertainty introduced by “DP”, so the trend of combined uncertainty of gas flowrate $u_{rel}\left(Q_{g}\right)$ is dominated by the component of “Cap”. Meanwhile, at certain liquid flowrate $Q_{l}$, with the gas flowrate $Q_{g}$ increasing, the component by “Cap” gradually increases whilst the component by “DP” gradually decreases, which causes the combined uncertainty of the gas flowrate $u_{rel}\left(Q_{g}\right)$ to decrease slightly at first and then increase gradually. With the liquid flowrate $Q_{l}$ increasing, the combined uncertainty of the gas flow rate $u_{rel}\left(Q_{g}\right)$ decreases monotonically. Therefore, the relative uncertainty of the gas flowrate $u_{rel}\left(Q_{g}\right)$ of the “DP + Cap” method reaches its maximum at low liquid flowrate $Q_{l}$ and large gas flowrate $Q_{g}$, and its contour is shown in Figure 12b.

#### 3.2.3. The Connections between Error and Uncertainty

**Figure 13:**
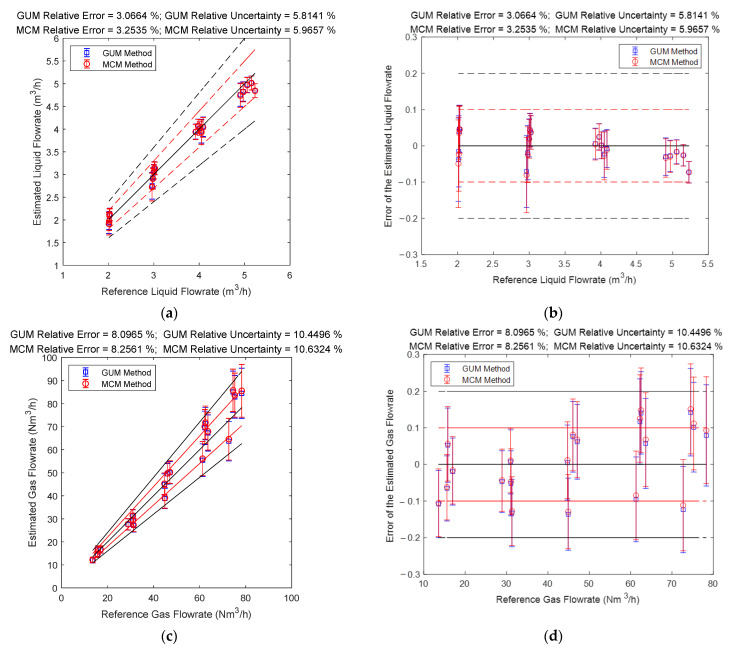
The prediction results of the liquid and gas flowrates of the “DP + Cap” method: (**a**) absolute error of the liquid flowrate; (**b**) absolute error of the liquid flowrate; (**c**) absolute error of the gas flowrate; (**d**) relative error of the gas flowrate. (The central black line denotes the ideal case with zero error, the upper and lower red lines denote the 10% relative error range, and the upper and lower black lines denote the 20% relative error range. The line segments denote the standard uncertainty whereas the distance between the point and the central black line denote the error. More information can be found in Section 2.4.2).

The gas and liquid flowrate prediction results of the “DP + Cap” method are shown in Figure 11. The blue lines in Figure 13 denote the GUM results, whilst the red lines denote the MCM results. It can be noted from Figure 13 that the GUM and MCM results generally overlap but discrepancy still exists, and the reasons for this discrepancy will be analyzed later in this section. From Figure 13, it can also be noted that the “DP + Cap” method has better prediction results for the liquid flowrate, especially for conditions with large liquid flowrate, whilst the prediction results for the gas flowrate is relatively poor, especially for conditions with large gas flowrate.

#### 3.2.4. The Discrepancies between GUM and MCM Results

**Figure 14:**
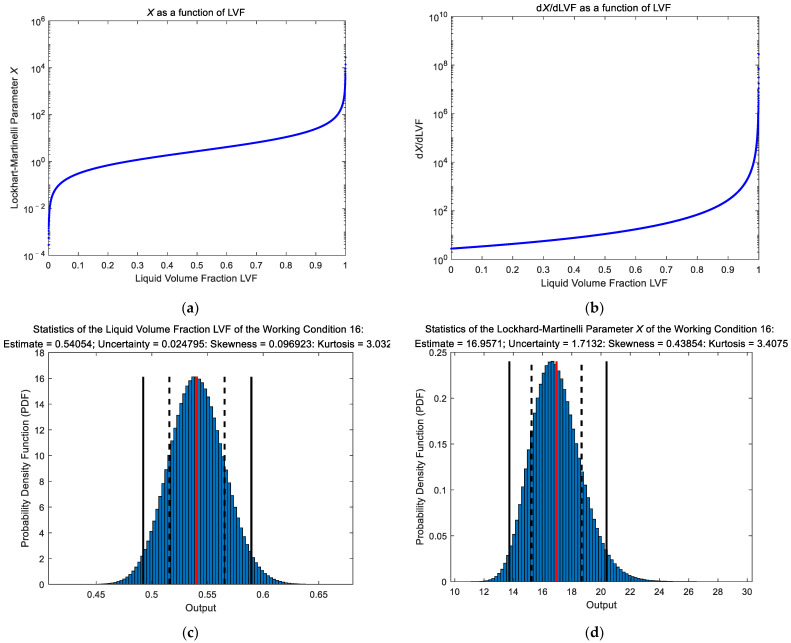
The change of the probability density function (PDF) of the “DP + Cap” method when the liquid volume fraction LVF is converted to the L-M number X: (**a**) X as a function of LVF; (**b**) dX/dLVF as a function of LVF; (**c**) PDF of a certain LVF; (**d**) PDF of the corresponding X.

From Figure 11b and Figure 12b, it can be noted that the GUM and MCM results of the “DP + Cap” method do not match very well. This is because when the liquid volume fraction LVF is converted to the L-M number X (Equation (13)), its range changes from [0,1] to [0,+∞) and its probability density function (PDF) begins to deviate from the normal distribution, as shown in Figure 14c,d. Figure 14a,b shows X and dX/dLVF as functions of the LVF, from which it can be noted that for a certain LVF, the increase of X caused by a positive perturbation of LVF is always larger than the decrease of X caused by a negative perturbation of LVF. Therefore, X is always right skewed and the larger LVF is, the more right-skewed X will become due to the increasing dX/dLVF. In this case, the gas/liquid flowrate output will also deviate from the normal distribution, and the MCM results should by adopted rather than the GUM ones in this case.

### 3.3. “CC + DP” Method

#### 3.3.1. The Calculation Procedures

**Figure 15:**
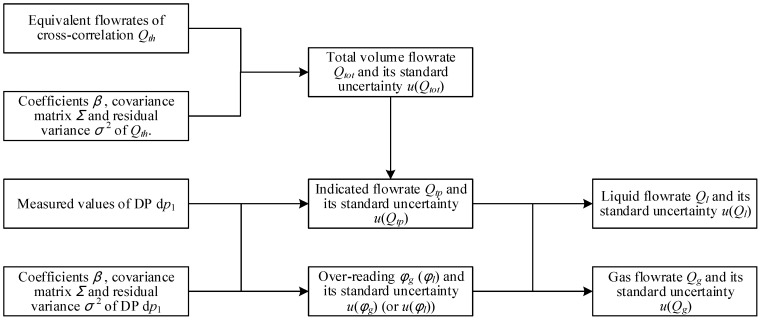
The calculation procedures of the cross-correlation and differential pressure (CC + DP) method.

The calculation procedure of the “CC + DP” method is shown in Figure 15. The method to calculate the estimate and standard uncertainty of $Q_{\mathrm{tot}}$ is exactly the same as the “Cap + CC” method, and the method to calculate the indicated gas flowrate $Q_{tp}$ and its uncertainty $u\left(Q_{tp}\right)$, coefficient $\hat{\beta }$ and covariance matrix $\Sigma_{\hat{\beta }\hat{\beta }}$ is exactly the same as the “DP + Cap” method.

After the estimates of the total volume flowrate $Q_{tot0}$ are obtained, $Q_{l}=Q_{tot}-Q_{g}$ is substituted into Equation (16) and then simplified as follows:

(19) $$Q_{g}=\frac{Q_{tp}-\beta_{1}\sqrt{\frac{\rho_{l}}{\rho_{g}}}Q_{tot}}{\beta_{0}-\beta_{1}\sqrt{\frac{\rho_{l}}{\rho_{g}}}}=\frac{Q_{tp}^{*}}{\phi_{g}^{*}}$$

Similarly, $Q_{g}=Q_{tot}-Q_{l}$ is substituted into Equation (16), and the liquid flowrate can be calculated by:

(20) $$Q_{l}=\frac{Q_{tp}-\beta_{0}Q_{tot}}{-\beta_{0}+\beta_{1}\sqrt{\frac{\rho_{l}}{\rho_{g}}}}=\frac{Q_{tpl}^{*}}{\phi_{l}^{*}}$$

Taking the gas flowrate as an example, the standard uncertainty of $Q_{tp}^{*}$ and $\phi_{g}^{*}$ can be derived from Equation (A8).

(21) $$u\left(Q_{tp}^{*}\right)=\sqrt{u^{2}\left(Q_{tp}\right)+\frac{\rho_{l}}{\rho_{g}}Q_{tot}^{2}u^{2}\left(\beta_{1}\right)+\frac{\rho_{l}}{\rho_{g}}\beta_{1}^{2}u^{2}\left(Q_{tot}\right)}$$

(22) $$u\left(\phi_{g}^{*}\right)=\sqrt{u^{2}\left(\beta_{0}\right)+\frac{\rho_{l}}{\rho_{g}}u^{2}\left(\beta_{1}\right)-2\sqrt{\frac{\rho_{l}}{\rho_{g}}}u\left(\beta_{0},\;\beta_{1}\right)}$$

The standard uncertainty involved in Equations (21) and (22) can be obtained from the covariance matrix $\Sigma_{\hat{\beta }\hat{\beta }}$ and the standard uncertainty u(y) of the dependent variable y.

When calculating the standard uncertainty of gas flowrate $\;u\left(Q_{g}\right)$, special attention should be paid to the correlation between $Q_{tp}^{*}$ and $\phi_{g}^{*}$.

(23) $$u\left(Q_{g}\right)=\frac{1}{\phi_{g}^{*2}}\sqrt{\phi_{g}^{*2}u^{2}\left(Q_{tp}^{*}\right)+Q_{tp}^{*2}u^{2}\left(\phi_{g}^{*}\right)-2\phi_{g}^{*}Q_{tp}^{*}u\left(\phi_{g}^{*},\;Q_{tp}^{*}\right)}$$

where:

(24) $$u\left(\phi_{g}^{*},\;Q_{tp}^{*}\right)=-\sqrt{\frac{\rho_{l}}{\rho_{g}}}Q_{tot}u\left(\beta_{0},\;\beta_{1}\right)+\frac{\rho_{l}}{\rho_{g}}Q_{tot}u^{2}\left(\beta_{1}\right)$$

The standard uncertainty of liquid flowrate $u\left(Q_{l}\right)$ can be calculated from Equation (20), with reference to Equations (21)–(24).

#### 3.3.2. The Composition and Distribution Diagrams

**Figure 16:**
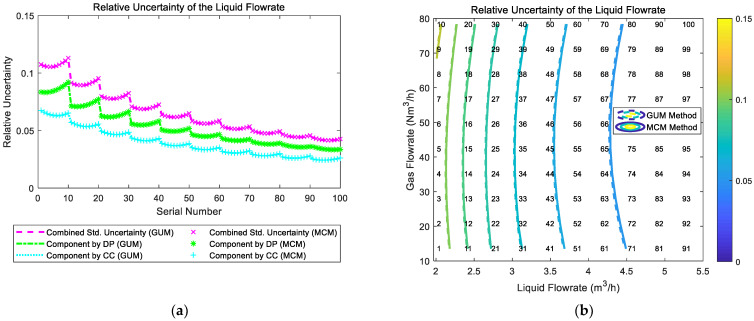
Composition and distribution of the relative uncertainty of the liquid flowrate of the “CC + DP” method: (**a**) composition diagram; (**b**) distribution diagram. (Each contour line denotes an increment of uncertainty of 0.01. More information about the composition and distribution can be found in Section 2.4.1).

The composition and distribution of the relative uncertainty of the liquid flowrate of the “CC + DP” method are shown in Figure 16. It can be noted from Figure 16a that the component of uncertainty introduced by “DP” and the component of uncertainty introduced by “CC” are relatively similar. In addition, at certain liquid flowrate $Q_{l}$, with the increasing of gas flowrate $Q_{g}$, the components by “DP” and “CC” do not change significantly, which causes the combined uncertainty of the liquid flowrate $u_{rel}\left(Q_{l}\right)$ to remain constant. With the increasing of liquid flowrate $Q_{l}$, the combined uncertainty of the liquid flowrate $u_{rel}\left(Q_{l}\right)$ decreases monotonically. Therefore, the contour of the relative uncertainty of liquid flowrate $u_{rel}\left(Q_{l}\right)$ of the “CC + DP” method is shown in Figure 16b.

**Figure 17:**
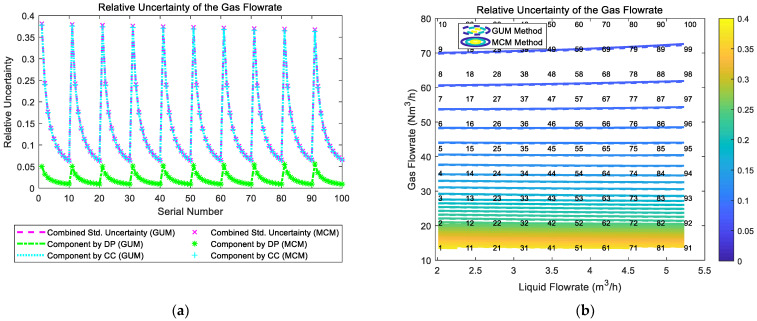
Composition and distribution of the relative uncertainty of the gas flowrate of the “CC + DP” method: (**a**) composition diagram; (**b**) distribution diagram. (Each contour line denotes an increment of uncertainty of 0.01. More information about the composition and distribution can be found in Section 2.4.1).

The composition and distribution of the relative uncertainty of the gas flowrate of the “CC + DP” method are shown in Figure 17. It can be noted from Figure 17a that the component of uncertainty introduced by “CC” is much larger than the component of uncertainty introduced by “DP”, so the trend of the combined uncertainty of gas flowrate $u_{rel}\left(Q_{g}\right)$ is dominated by the “CC” component. In addition, at certain liquid flowrate $Q_{l}$, with the increasing of gas flowrate $Q_{g}$, the components by “CC” and “DP” decrease rapidly, which causes the combined uncertainty of gas flowrate $u_{rel}\left(Q_{g}\right)$ to decrease rapidly. With the increasing of liquid flowrate $Q_{l}$, the combined uncertainty of the gas flowrate $u_{rel}\left(Q_{g}\right)$ generally does not change. Therefore, the contour of the relative uncertainty of gas flowrate $u_{rel}\left(Q_{g}\right)$ of the “CC + DP” method is shown in Figure 17b.

#### 3.3.3. The Connections between Error and Uncertainty

**Figure 18:**
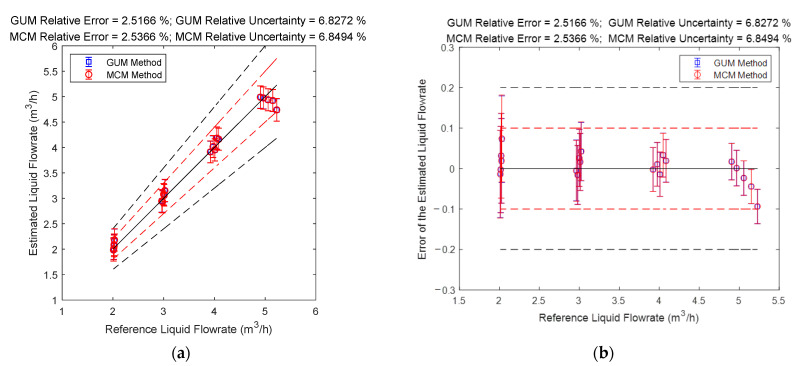

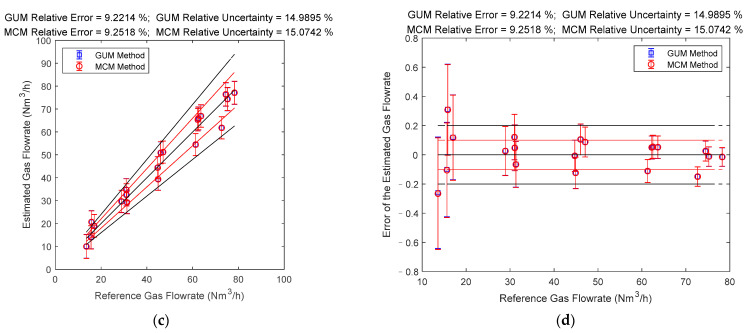
The prediction results of the liquid and gas flowrate of the “CC + DP” method: (**a**) absolute error of the liquid flowrate; (**b**) relative error of the liquid flowrate; (**c**) absolute error of the gas flowrate; (**d**) relative error of the gas flowrate. (The central black line denotes the ideal case with zero error, the upper and lower red lines denote the 10% relative error range, and the upper and lower black lines denote the 20% relative error range. The line segments denote the standard uncertainty whereas the distance between the point and the central black line denote the error. More information can be found in Section 2.4.2).

The gas and liquid flowrate prediction results of the “CC + DP” method are shown in Figure 18. It can be noted from Figure 18 that the liquid flowrate prediction results of the “CC + DP” method are very stable and accurate, especially for conditions with high liquid flowrates. By contrast, the gas flowrate prediction results of this algorithm is very unstable, and its accuracy decreases rapidly with the gas flowrate decreasing, which results in good results at large gas flowrate but bad results at low gas flowrate. Therefore, this algorithm is more suitable for conditions with large gas/liquid flowrates.

## 4. Multi-Sensor Fusion Algorithm

### 4.1. The Calculation Procedures

**Figure 19:**
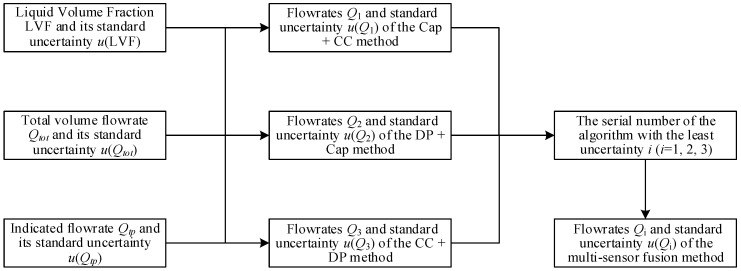
The calculation procedures of the multi-sensor fusion algorithm.

The calculation procedures of the multi-sensor fusion algorithm are shown in Figure 19. Its basic idea is to calculate the flowrate estimates and associated standard uncertainties of the above three common algorithms, and then record the serial number of the method with the least uncertainty. Finally, the results of the algorithm with the recorded serial number will be outputted as the final results, as shown in Figure 19.

### 4.2. The Composition and Distribution Diagrams

**Figure 20:**
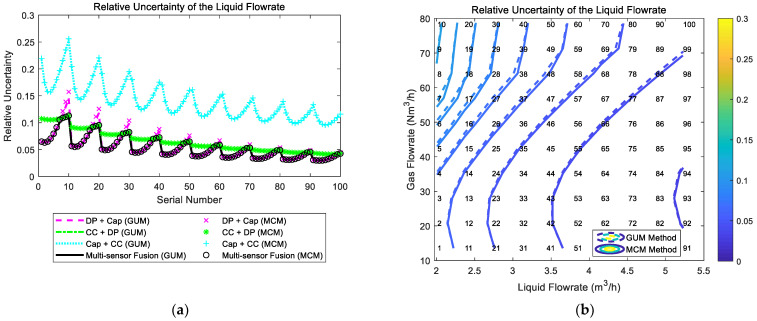
The composition and distribution of the relative uncertainty of the liquid flowrate of the multi-sensor fusion algorithm: (**a**) composition diagram; (**b**) distribution diagram. (Each contour line denotes an increment of uncertainty of 0.01. More information about the composition and distribution can be found in Section 2.4.1).

The composition and distribution of the relative uncertainty of the liquid flowrate of the multi-sensor fusion algorithm are shown in Figure 20. It can be noted from Figure 20a that the uncertainty of the “DP + Cap” method and the uncertainty of the “CC + DP” method are relatively similar, whilst the uncertainty of the “Cap + CC” method is inferior to the other two. In addition, at certain liquid flowrate $Q_{l}$, with the gas flowrate $Q_{g}$ increasing, the uncertainty of the “DP + Cap” method gradually increases, whilst the uncertainty of the “CC + DP” method slightly decreases. Therefore, the uncertainty of the multi-sensor fusion algorithm is essentially a combination of the “DP + Cap” method at low gas flowrate conditions and the “CC + DP” method at high gas flowrate conditions. With the liquid flowrate $Q_{l}$ increasing, the uncertainties of all three algorithms decrease monotonically, so the contour of the relative uncertainty of multi-sensor fusion algorithm is shown in Figure 20b.

**Figure 21:**
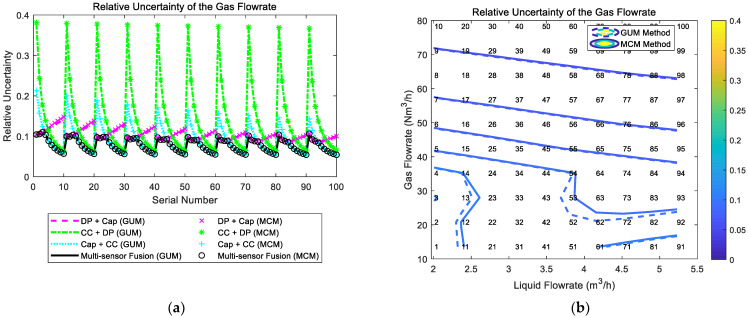
The composition and distribution of the relative uncertainty of the gas flowrate of the multi-sensor fusion algorithm: (**a**) composition diagram; (**b**) distribution diagram. (Each contour line denotes an increment of uncertainty of 0.01. More information about the composition and distribution can be found in Section 2.4.1).

The composition and distribution of the relative uncertainty of the gas flowrate of the multi-sensor fusion algorithm are shown in Figure 21. It can be noted from Figure 21a that the uncertainty of the “DP + Cap” method and the uncertainty of the “Cap + CC” method are relatively similar, whilst the uncertainty of the “CC + DP” method is inferior to the other two. In addition, at certain liquid flowrate $Q_{l}$, with the gas flowrate $Q_{g}$ increasing, the uncertainty of the “DP + Cap” method gradually increases, whilst the uncertainty of the “Cap + CC” method rapidly decreases. Therefore, the gas flowrate uncertainty of the multi-sensor fusion algorithm is essentially a combination of the “DP + Cap” method under low gas flowrate conditions and “Cap + CC” method under large gas flowrate conditions. With the liquid flowrate $Q_{l}$ increasing, the combined uncertainties of the gas flowrate of the three algorithms decrease monotonically to a certain extent, so the contour of the gas flowrate relative uncertainty of multi-sensor fusion algorithm is shown in Figure 21b.

### 4.3. The Connections between Error and Uncertainty

**Figure 22:**
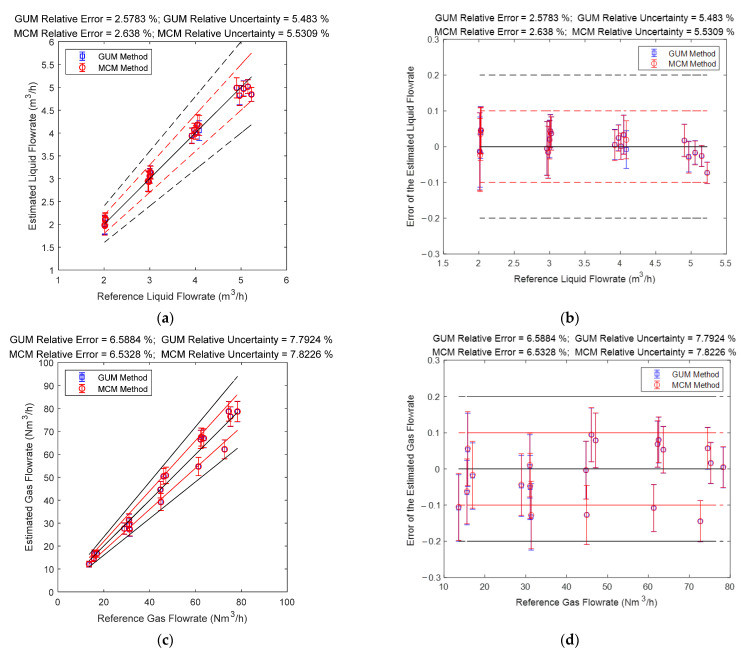
The prediction results of the gas and liquid flowrates of the multi-sensor fusion algorithm: (**a**) absolute error of the liquid flowrate; (**b**) relative error of the liquid flowrate; (**c**) absolute error of the gas flowrate; (**d**) relative error of the gas flowrate. (The central black line denotes the ideal case with zero error, the upper and lower red lines denote the 10% relative error range, and the upper and lower black lines denote the 20% relative error range. The line segments denote the standard uncertainty whereas the distance between the point and the central black line denote the error. More information can be found in Section 2.4.2).

The gas and liquid flowrate prediction results of the multi-sensor fusion algorithm are shown in Figure 22. It can be noted from Figure 22 that the liquid flowrate prediction results of the multi-sensor fusion algorithm are very stable and accurate, especially at conditions with large liquid flowrate. Meanwhile, the gas flowrate prediction results of this algorithm are also very stable and accurate, especially at conditions with large gas flowrate. From Figure 22, it can also be noted that the multi-sensor fusion algorithm enjoys much lower uncertainty and error than any of the three conventional algorithms, which demonstrates that uncertainty analysis can be used for improving the accuracy of a MPFM.

In theory, the multi-sensor fusion algorithm can combine the results of as many conventional algorithms as possible to increase its accuracy. In practice, most multiphase flowmeters have sensor redundancy, so it is possible that there are several available algorithms for one working condition, and the multi-sensor fusion algorithm can take advantage of this situation by just outputting the one will the least uncertainty and improve the system accuracy as a result.

## 5. Conclusions

In this paper, three common combinations of multiphase flow sensing methods (“Cap + CC”, “CC + DP” and “DP + Cap”) are implemented for uncertainty analysis and the analytical expressions of the liquid/gas flowrates and associated standard uncertainty are derived. Meanwhile, Monte Carlo simulations are conducted to determine the PDF of the gas and liquid flowrate. The results obtained through these two approaches are generally the same, such as the estimates and standard uncertainties. In addition, the following important conclusions can be obtained through this research.

1. The standard uncertainties of each algorithm are different under different flow conditions. Even the standard uncertainties of different algorithms differ from each other under the same flow conditions (e.g., the difference in distributions are clearly shown in Figure 6, Figure 7, Figure 11, Figure 12, Figure 16, Figure 17, Figure 20 and Figure 21). The difference in distributions can provide a basis for a multi-sensor fusion algorithm: if there are multiple available algorithms for one flow condition, the multiphase flowmeter can simply output the results of the algorithm with the lowest uncertainty in order to reduce the measurement error.
2. The percentages of uncertainty introduced by different sensors are also different, and these percentages vary with the flowrates (e.g., in Figure 7a component by “CC” is much larger than component by “Cap”, but they are similar in Figure 6a). This conclusion can help reveal the composition characteristics of different algorithms, and provide guidance for sensor selection and algorithm development.
3. The level of uncertainty can be used for error prediction (e.g., the MAPE and MAPU results presented in the titles of Figure 8, Figure 13, Figure 18 and Figure 22 are similar), but relevant uncertainty analysis is often absent in the literature. The analysis in this paper can help the operators better know the accuracy of their measurements, thereby providing important guidance for MPFM calibrations.
4. The uncertainties obtained through the GUM and MCM approaches are generally the same (e.g., the difference between GUM and MCM results in Figure 6, Figure 7, Figure 11, Figure 12, Figure 16, Figure 17, Figure 20 and Figure 21 are negligible). With the assistance of advanced statistical and simulation software, the MCM approach can be used directly for more complicated algorithms, such as those with non-linear fittings and iterations, whilst the GUM approach can be used to verify the results of MCM approach under certain simplified conditions.

## Data Availability

The data presented in this study are available on request from the corresponding author.

## Nomenclature

**Variables:**	
*A*	Cross-sectional Area of the Venturi throat
*A_d_*	Cross-sectional Area of the pipe
*C*	Capacitance
*C_d_*	Discharge coefficient
*c*	Sensitivity coefficients
*D*	Diameter of the Venturi throat
*d*	Diameter of the pipe or Venturi inlet
*dp*	Differential pressure
*dt*	Time interval between two detection of the dual-ECT
*e*	Error term
*f*	Detection frequency or function
*L*	Distance between the two planes of dual-ECT
*M*	Number of simulation tests
*n*	Total number or serial number
*p*	Pressure or level of confidence
*Q*	Volume flowrate
*Q_tot_*	Total Volume flowrate
*Q_th_*	Estimated total flowrate of the dual-ECT
*Q_tp_*	Indicated gas flowrate of the Venturi
*s*	Standard deviation
*T*	Temperature
*t*	time
*U*	Expanded uncertainty
*u*	Standard uncertainty
*u_rel_*	Relative standard uncertainty
*u_c_*	Combined uncertainty
*X*	Lockhart-Martinelli Parameter
*x*	Independent variable
*Y*	The reciprocal of *X*
*y*	Measurand or dependent variable
**Symbols:**	
*β*	Diameter ratio or regression coefficients
*ε*	Expansion factor
Σ	Covariance Matrix
*σ^2^*	Residual Variance
*ρ*	Density
*τ*	Time delay
*φ*	Over-reading
**Abbreviations:**	
Cap	Capacitance
CC	Cross-Correlation
DP	Differential Pressure
ECT	Electrical Capacitance Tomography
ERT	Electrical Resistance Tomography
Eq.	Equation
Fig.	Figure
GUM	Guide to the expression of Uncertainty in Measurement
L-M	Lockhart-Martinelli Parameter
LVF	Liquid Volume Fraction
MAE	Mean Absolute Error
MAPE	Mean Absolute Percentage Error
MAPU	Mean Absolute Percentage Uncertainty
MAU	Mean Absolute Uncertainty
MCM	Monte Carlo Method
MPFM	Multiphase Flow Meter
PDF	Probability Density Function
Std.	standard
UA	Uncertainty Analysis
**Subscripts:**	
avg	average
g	gas
l	liquid
rel	relative
0	Estimated value

## Appendix A

### Appendix A.1. Evaluating Input Uncertainty

There are two approaches to evaluate the standard uncertainty of the input quantities: *Type A* evaluation based on repeated observations and *Type B* evaluation based on other available information [38]. For all algorithms in this paper, the direct measurements are first converted into an important intermediate variable (e.g., liquid volume fraction LVF or total volume flow rate $Q_{\mathrm{tot}}$) through the least-squares method, and then these intermediate variables are combined to calculate the gas/liquid flowrate outputs. Therefore, the source of uncertainty can only come from the linear regression.

Linear regression model assumes that the observed value of $Y_{i}$ is a linear function of $x_{ij}$ plus a disturbance term [45]:

(A1) $$Y_{i}=\beta_{0}+\overset{p-1}{\underset{j=1}{\sum }}\beta_{j}x_{ij}+e_{i},i=1,\ldots ,n$$

where $e_{i}$ is a random error variable, with $E\left(e_{i}\right)=0$, $\mathrm{Var}\left(e_{i}\right)=\sigma^{2}$, and $\mathrm{Cov}\left(e_{i},e_{j}\right)=0$, i≠j.

Using matrix notation, Equation (A1) can be written as:

(A2) $$Y_{n\times 1}=X_{n\times p}\beta_{p\times 1}+e_{n\times 1}$$

where E(e)=0, $\Sigma_{ee}=\sigma^{2}I$.

It can be proved that the best unbiased estimates $\hat{\beta }$ of the coefficients β and the associated covariance matrix $\Sigma_{\hat{\beta }\hat{\beta }}$ are respectively:

(A3) $$\hat{\beta }={\left(X^{T}X\right)}^{-1}X^{T}Y$$

(A4) $$\Sigma_{\hat{\beta }\hat{\beta }}=\sigma^{2}{\left(X^{T}X\right)}^{-1}$$

where $\sigma^{2}$ is the expected value of the square of the error $e_{i}$, and it can be proved that the best unbiased estimate of $\sigma^{2}$ is:

(A5) $$s^{2}=\frac{\left||Y-{\hat{Y}}^{2}|\right|}{n-p}$$

To sum up, the least squares model assumes that the independent variable X is not a random variable, but a constant that can be precisely measured and controlled, whilst the dependent variable Y always has a constant variance $\sigma^{2}$ due to the impact of the error term e**.** The specific expression of the coefficients $\hat{\beta }$ and covariance matrix $\Sigma_{\hat{\beta }\hat{\beta }}$ for each algorithm can be derived by Equations (A3)–(A5).

### Appendix A.2. Determining Output Uncertainty

If the measurement model can be expressed as follows:

(A6) $$Y=f\left(X\right)=f\left(X_{1},\cdots ,X_{N}\right)$$

Then the estimate of its output is:

(A7) $$y=f\left(x_{1},\cdots ,x_{N}\right)$$

and the combined standard uncertainty $u_{c}\left(y\right)$ of the output estimate y can be determined through the following equation:

(A8) $$u_{c}^{2}\left(y\right)=\overset{n}{\underset{i=1}{\sum }}\overset{n}{\underset{j=1}{\sum }}\frac{\partial f}{\partial x_{i}}\frac{\partial f}{\partial x_{j}}u\left(x_{i},x_{j}\right)=c^{T}\Sigma_{x}c$$

This equation is also referred to as the law of propagation of uncertainty. In Equation (A8) $u\left(x_{i},x_{j}\right)$ is the covariance between the input quantities $x_{i}$ and $x_{j}$, and $\Sigma_{x}$ is the corresponding covariance matrix. If the input quantities $x_{i}$ and $x_{j}$ are coefficients obtained through least squares fitting, then $\Sigma_{x}=\Sigma_{\hat{\beta }\hat{\beta }}$. $\frac{\partial f}{\partial x_{i}}$ is the sensitivity coefficient, usually obtained by calculating the partial derivative of the function f at $x_{i}$, $c^{T}=\left[\frac{\partial f}{\partial x_{1}},\cdots ,\frac{\partial f}{\partial x_{n}}\right]$ is a vector of the sensitivity coefficients. If the measurement model is complicated and the partial derivatives are difficult to calculate, then $\frac{\partial f}{\partial x_{i}}$ can also be determined through numerical or experimental methods.
